# Antiviral Therapy in HCV-infected Decompensated Cirrhotics

**DOI:** 10.4103/1319-3767.70632

**Published:** 2010-10

**Authors:** Fazal A. Danish, Salman S. Koul, Fazal R. Subhani, Ahmed E. Rabbani, Saeeda Yasmin

**Affiliations:** St Mary’s Hospital, Isle of Wight, PO30 5TG, UK; 1 Department of Medicine, Pakistan Institute of Medical Sciences (PIMS), Islamabad, Pakistan; 2 Department of Pediatrics, Holy Family Hospital, Rawalpindi, Pakistan; 3 Foundation University Medical College (FUMC), Rawalpindi, Pakistan; 4 Department of Surgery, Shifa International Hospital, Islamabad, Pakistan

**Keywords:** Antiviral therapy, chronic hepatitis C, decompensated cirrhosis, hematopoietic growth factors

## Abstract

Decompensated cirrhosis has traditionally been considered a contraindication to interferon and ribavirin therapy. Whereas, the same may be true for advanced cirrhosis, which is only successfully amenable to liver transplantation (LT), there are reports in the literature in which antiviral therapy was given successfully in selected cases of early hepatic decompensation with an aim to attain sustained viral clearance, halt disease progression, and expect potential (though, often, partial) recovery of hepatic metabolic activity. Antiviral therapy may also be instituted to prevent hepatitis C recurrence after LT (it has even caused removal of some patients from the waiting list for LT). Thus, decompensation per se is no more an absolute contraindication to antiviral therapy. Nonetheless, considering that a large proportion of such patients have pre-existing hematological cytopenias, modifications in antiviral dose regimens and close monitoring is required in order to prevent worsening of the same. Although the final sustained virological response rates attained in these patients are relatively low, successful antiviral therapy is potentially lifesaving which explains the need to go for it. In this article, the pros and cons of antiviral therapy in decompensated liver cirrhosis are reviewed with special emphasis on how to avoid antiviral dose reductions/withdrawals secondary to the development of hematologic side effects by using hematopoietic growth factors.

Whereas, decompensated cirrhosis of liver has traditionally been considered a contraindication to antiviral therapy, the same is not true anymore. Ribavirin-induced hemolytic anemia and interferon-induced neutropenia are one of the most common causes of antiviral dose reductions/withdrawal, particularly in decompensated cirrhotics. Although, no consensus still exists, there are some recent reports in the literature that suggest the use of hematopoietic growth factors (HGF’s) in selected cases of hemolytic anemia and neutropenia. Whereas, the addition of growth factors substantially increases the overall cost of the treatment, the same have provided an opportunity to institute antiviral therapy in some of the conditions previously included in the list of contraindications to antiviral therapy (like decompensated cirrhosis). Although more studies are needed to truly define the indications, dose regimen, side effects, and therapeutic efficacy of these factors, the initial results are encouraging and hematopoietic growth factors appear to be a useful adjunct to the antiviral therapy.

Fibrosis is the histopathological hallmark of chronic hepatitis causing progressive derangement of normal liver architecture with consequent reduction in hepatic synthetic function. Chronic liver disease is said to be decompensated when one or the other complication of chronic liver disease has developed - ascites, variceal bleeding (secondary to portal hypertension), impaired hepatic synthetic function (hypoalbuminemia), jaundice, or hepatic encephalopathy. Five year survival rate in decompensated cirrhotics is estimated to be 50%.[1] Liver transplantation (LT) is the treatment of choice in all such cases. If hepatitis C virus (HCV) is not eradicated before going for LT, reinfection with HCV occurs in *all* transplant recipients as a rule. This in turn leads to cirrhosis in around 30% patients in 5 years.[2] It is thus very common to see progressive post-transplantation disease of the allograft in HCV-infected cases. Pre-transplantation HCV eradication is associated with less likelihood of reinfection and this forms the rationale for treating decompensated cirrhotics waiting LT with antiviral therapy;[3] initiating pre-emptive post-transplantation antiviral therapy, and treating established post-transplant chronic hepatitis being other therapeutic options in cirrhotics.

LT per se is not a practical option for a great majority of the cirrhotic patients. This is not only because of limited number of organ donors available at a given time, but also because of the age-related cardiovascular, renal and/or pulmonary derangements that practically make going for LT infeasible and at times rather irrational. Additionally, old age (≥65 years) is generally considered an exclusion criterion for LT. In a nutshell, exploring and offering some potentially successful treatment option (like antiviral therapy) is the need of the hour in cirrhotics.

The aim of instituting pre-transplantation antiviral therapy is either to attain a SVR at transplantation, or an on-treatment HCV RNA clearance at transplantation. Importantly, mere reduction of viral load should not be the aim because, unlike HBV cirrhotics, this has not been shown to decrease the rate and/or severity of recurrence in HCV cases.

Traditionally, despite the known theoretical benefits of antiviral therapy (improvement in liver histology, partial reversal of established cirrhosis, and prevention of life-threatening complications), many cirrhotic patients have not been offered antiviral therapy. Peginterferon-ribavirin combination therapy has limited efficacy in patients with decompensated cirrhosis.[4,5] Also, antiviral therapy is not safe from potentially serious adverse effects in this population group. As decompensated cirrhotics are more prone to develop hematologic side effects (neutropenia, thrombocytopenia and anemia) with antiviral therapy as compared to non-cirrhotics,[6] patients who already have neutropenia or thrombocytopenia below the permissible limits (neutrophil count >1500/mm^3^; thrombocyte count >75,000/mm^3^) are highly prone to develop life-threatening infections after starting antiviral therapy, particularly if they have Child-Pugh class C disease.[7,8] Also, it is generally thought that age-related derangements in cardiovascular and pulmonary functions make the cirrhotic patients less tolerant to ribavirin-induced hemolytic anemia. Finally, there are concerns regarding decompensation actually made worse by antiviral therapy as is the case with decompensated chronic hepatitis B cases[9] (if you can’t do any good to the patient, at least don’t harm him, policy!).

The current literature review, however, shows that because of the unstandardized dosage schedules being administered over variable periods of time in the past studies, we have under- and overestimated the potential benefits and risks of antiviral therapy respectively in decompensated cirrhotic patients. There are now several reports in the literature in which antiviral therapy was relatively well tolerated in decompensated cirrhotic patients with reasonable attainment of ETR and SVR rates.[4,7,10,11] In one study,[7] 39% of the patients receiving low, accelerating regimen of non-pegylated interferon plus ribavirin experienced clearance of HCV-RNA, and 21% attained an SVR. Results with pegylated interferon are even better. In the first study[12] proving the benefits of antiviral therapy in cirrhotics with signs of portal hypertension, 51 cirrhotics received 1 mg/kg/week of pegylated-interferon α-2b plus oral ribavirin at a fixed dose of 800 mg/day for 52 weeks. By intention-to-treat analysis, SVR was achieved in 21.6% patients. As otherwise, patients with genotypes 2 and 3 showed better results (83.3%) than genotype 1 cases (13.3%). Although antiviral therapy was stopped in five of the patients because of neutrophil counts falling below 0.75×10^3^/dL, none of them developed superadded infections. The disease deteriorated in only 6% of those who attained SVR compared to 38% of the non-responders. In another study,[10] Peg-IFN α-2b (1.0 mg/kg) plus standard dose of ribavirin were administered to all patients for 24 weeks regardless of the genotype. The overall SVR rate attained even with this suboptimal dose regimen was 19.7%. Except patients with very advanced liver disease (CTP score >10), none experienced life-threatening complications. Peg-IFN and ribavirin in the standard dosage (Peg-IFN α-2b 1.5 mg/kg and ribavirin 800-1000 mg for genotypes 2 and 3, and 1000-1200 mg for genotypes 1 and 4) for the standard duration of time (48 and 24 weeks for genotype 1 and non-1, respectively) have also been tried. In one study,[13] 35% of end-staged cirrhotics cleared the HCV infection (16% genotype 1 and 4, and 59% genotype 2 and 3 cases). 60% of all patients tolerated this ‘standard’ treatment without any major untoward effect; treatment was discontinued in 19.1% of the patients with 4 among those ending up having severe superadded infections. In yet another study,[14] a 48 week course was planned for patients who demonstrate EVR with a standard regimen of PEG-IFN alfa-2a (135*µ*g, once a week) plus ribavirin (1000-1200 mg/day). Results showed 60% patients completing the course, with ETR and SVR achieved in 45% and 35% cases respectively. In a recent study,[15] aimed to evaluate both the prevention of post-transplantation HCV recurrence and the risk of bacterial infections during therapy, 47% patients achieved HCV RNA negativity during treatment, 29% were HCV RNA negative at the time of transplantation (drop outs n=3, deaths n=4, viral relapse n=2), and 20% achieved an SVR post-transplantation. Importantly, none of the patients who achieved SVR pre-transplantation developed a recurrence post-transplantation.

Based on the current literature review it is suggested that all cirrhotic patients with a CTP score ≤9 and history of a decompensated event that abated with routine therapy be offered antiviral therapy. A suggested protocol could be Peg-IFN α-2b in a dose of 1.5 mg/kg and ribavirin in a dose of 800-1000 mg for genotypes 2 and 3, and 1000-1200 mg for genotypes 1 and 4 for 48 and 24 weeks for genotype 1 and non-1, respectively. As otherwise, attainment of a rapid / early virological response and genotypes 2 and 3 are the most robust predictors of viral clearance with antiviral therapy.[10,12] Child-Pugh score class A (in genotype 1 cases only) and lower pre-transplantation viral loads are other positive predictors. A reduction in the viral load of ≤2 log_10_between baseline and week 4, and baseline Child-Pugh score of C or MELD >18 have a strong negative predictive value. In the absence of a ≥2 log_10_reduction in HCV RNA at week 4, probably the best approach to reduce the risk of complications is to stop antiviral therapy at this point.

As a general rule, decompensated cirrhotics are more prone to develop drug-induced side-effects compared to patients with compensated disease. Drug-induced neutropenia, thrombocytopenia, anemia, superadded infections (SBP etc), and liver decompensation during therapy are reported to occur in 50-60%, 30-50%, 30-60%, 4-13%, and 11-20% of decompensated cirrhotic cases respectively.[4,10,12] In one study,[16] the relative frequencies of clinical decompensation (22% vs. 18%, *P*=0.62), death before LT (8% vs. 2% (*P*=0.06) or 24 weeks after LT (8% vs. 12%; (*P*=0.67) were similar in treated and control subjects. However, a significantly higher incidence of superadded infections (spontaneous bacterial peritonitis and spontaneous bacteremia due to Gram-negative bacilli) was noted in the treated subjects (25%) compared to the controls (6%) (*P*=0.01). Septic shock developed in 10% of the treated subjects compared to none in the control arm (*P*=0.05). Studies have demonstrated that, besides antiviral therapy, variables independently associated with higher incidence of infective episodes include Child-Pugh class C (score of 12 (±1.2) and a neutrophil count <900 µL during treatment.[1,7] Norfloxacin prophylaxis has been shown to reduce the incidence of superadded infections.[15,16] In cases of established nosocomial SBP, multiresistant bacteria resistant to third-generation cephalosporins or amoxicillin-clavulanic acid are frequently found and should be treated with broad-spectrum antibiotics like carbapenems or glycopeptides.

The minimum effective doses of pegylated interferon and ribavirin appear to be 1 *µ*g/kg/week and 10.6 mg/kg/day, respectively. In case a hematologic side effect develops, it is recommended to first reduce the dose of the antiviral therapy to the minimum effective. If no or little improvement occurs in blood counts, use of hematopoietic growth factors (HGF’s) should be considered. HGF’s that include erythropoietin (EPO)[17,18] for ribavirin-induced hemolytic anemia, and granulocyte colony-stimulating-factor (G-CSF)/granulocyte monocyte-colony stimulating-factor (GM-CSF)[19,20] for interferon-induced leucopenia may be used with an aim to avoid dose reductions, something that compromises drug efficacy and possibly final SVR rates attained.

Possible indications of EPO include a fall in Hb level by >4 g/dL, Hb levels of <8 g/dL, and patients developing symptoms and signs of anemia (palpitations, dyspnea, easy fatigability, pallor).[21,22]

A suggested dose regimen for EPO is 20,000-40,000IU SQ weekly in three divided doses (max. 60,000IU/week) with an aim to maintain Hb level of >11 g/dL (return to the pretreatment level is not the aim).[23] Another study suggested starting EPO therapy at a lower dose of 4,000 IU SQ thrice weekly (12,000 IU/week) and then increasing the dose depending upon the response.[24] The first evidence of a response to the thrice weekly EPO administration is an increase in the reticulocyte count within 10 days.[25] Since erythroid progenitors take several days to mature, a clinically significant increase in hematocrit is usually not observed in less than 2 weeks and may require up to 6 weeks in some patients.[26] If the rate of rise of hemoglobin is greater than 1 g/dL over 2 weeks, it generally warrants decreasing EPO dose. This is because a greater than 1 g/dL rise in *any* 2 weeks during the course of the therapy has been associated with an increased risk of thromboembolic phenomenon, predisposing to myocardial infarction, stoke and even death.[27] Also, according to manufacturer’s recommendations, a Hb level of greater than 12 g/dL should not be aimed, the reason being potentially increased risk of thromboembolic phenomenon.[28] Once adequate Hb level (≥10 g/dL) is achieved, ribavirin dose can be increased to the optimum level.[20] Once started, adjunct EPO therapy may be required till the very end of the treatment. In one study,[24] the median duration of EPO treatment was 24 weeks (range 6-39).

Regarding G-CSF, the current recommendation[21] is to reduce IFN dose if neutrophil count falls to <0.5 × 1 0^9^/L, and discontinue it if it falls to <0.3 × 10^9^/L.[17] Regarding platelet count, IFN dose should be reduced if platelet count falls to <30 × 10^9^/L, and discontinued if it falls to <20 × 10^9^/L.[17] The minimum effective dose of pegylated interferon appears to be 1 µg/kg/week. If despite of reducing the pegylated interferon dose to the minimum effective level, neutrophil counts of <0.5 × 10^9^/L and platelet counts of <30 × 10^9^/L persist, instituting G-CSF therapy may be considered.[21]

A suggested dose regimen is to start G-CSF therapy at a dose of 30 MU SQ once weekly and then to adjust it as per the response/requirement. Complete blood counts should be asked twice or thrice weekly and response to therapy monitored. Once adequate neutrophil count is achieved, IFN dose can be increased to the optimum level.[21] Once started, adjunct G-CSF therapy may be required till the end of the treatment. In one study,[24] the median duration of G-CSF therapy was 20 weeks (range 9-45).

Although it is not yet clear how much survival benefit antiviral therapy confers, a standardized mortality rate analysis in one study reported a lower liver-related mortality among cirrhotics with SVR (0.6: CI: 0.0-3.1) compared to untreated patients.[29] In post-liver transplant cases, avoidance of allograft failure due to recurrence of HCV infection has also been reported in the literature although it needs further study and validation.[30]

## CONCLUSION

Although, decompensated cirrhosis of liver is no more considered an absolute contraindication to interferon therapy, because of the high risk of septic complications and low probability of attainment of an SVR, patients with Child-Pugh class C, CTP score ≥10 or MELD score 18 disease are not considered appropriate candidates for antiviral therapy. The ideal candidate for antiviral therapy remains a patient with Child-Pugh class A disease in whom the risk of drug-induced side effects is almost identical to that of the controls. Whether or not to institute antiviral therapy in Child-Pugh class B, patients should be individualized on case-to-case basis giving due consideration to factors like genotype and pre-treatment viral loads with antiviral therapy discontinued after 4 or 12 weeks if there is no virological response. Standard schedules of treatment may be considered in all patients with genotype 2 and 3 HCV infection; in genotype 1 cases, however, the risk-benefit ratio still needs to be defined. All cirrhotic patients on antiviral therapy need adjustment of the dosage schedule in accordance with the tolerability of the patient, especially in response to the development of hematologic side effects. HGF’s, though not routinely recommended, appear to be a useful adjunct to antiviral therapy to reduce antiviral dose reductions/withdrawal. Since, addition of HGF’s substantially increases the overall cost of the therapy, more studies are needed to establish the lower cut off limits for different blood counts below which HGF therapy may be considered. Additionally, norfloxacin prophylaxis has been shown to substantially reduce the risk of superadded infections. One thing that has increasingly become clear from the existing trial’s data is that cirrhotic patients who achieve SVR are less likely to develop liver-related complications as compared to the non-responders. Despite the many encouraging studies in the recent past, however, data on the long-term disease progression, avoidance of transplantation, and most importantly, improvement of life expectancy are still sparse. Although liver functions have clearly been shown to improve with antiviral therapy (as indicated by significant reductions in CTP and MELD scores), the same are more likely to deteriorate within a few years in patients with advanced cirrhosis thus explaining the need to accumulate data on the survival benefit conferred by antiviral therapy in cirrhotic patients. Although not yet tried, novel therapeutic strategies like direct antiviral agents are likely to be most beneficial in patients with decompensated disease.
